# Effects of Host Plants Reared under Elevated CO_2_ Concentrations on the Foraging Behavior of Different Stages of Corn Leaf Aphids *Rhopalosiphum maidis*

**DOI:** 10.3390/insects10060182

**Published:** 2019-06-23

**Authors:** Yu Chen, Clément Martin, Junior Corneille Fingu Mabola, François Verheggen, Zhenying Wang, KangLai He, Frederic Francis

**Affiliations:** 1 State Key Laboratory for Biology of Plant Disease and Insect Pests, Institute of Plant Protection, Chinese Academy of Agricultural Science, Beijing 100193, China; ychen_007@126.com (Y.C.); zywang@ippcaas.cn (Z.W.); hekanglai@caas.cn (K.L.H.); 2 Functional and Evolutionary Entomology, TERRA, Gembloux Agro-Bio Tech, Liège University, Passage des Déportés, 2, 5030 Gembloux, Belgium; cmartin@uliege.be (C.M.); jcfingu@doct.uliege.be (J.C.F.M.); fverheggen@uliege.be (F.V.)

**Keywords:** climate change, corn leaf aphid, foraging behavior, volatile organic compounds (VOCs)

## Abstract

Climate change is a major environmental concern and is directly related to the increasing concentrations of greenhouse gases. The increase in concentrations of atmospheric carbon dioxide (CO_2_), not only affects plant growth and development, but also affects the emission of plant organic volatile compounds (VOCs). Changes in the plant odor profile may affect the plant-insect interactions, especially the behavior of herbivorous insects. In this study, we compared the foraging behavior of corn leaf aphid (*Rhopalosiphum maidis*) on barley (*Hordeum vulgare* L.) seedlings grown under contrasted CO_2_ concentrations. During the dual choice bioassays, the winged and wingless aphids were more attracted by the VOCs of barley seedlings cultivated under ambient CO_2_ concentrations (aCO_2_; 450 ppm) than barley seedlings cultivated under elevated CO_2_ concentrations (eCO_2_; 800 ppm), nymphs were not attracted by the VOCs of eCO_2_ barley seedlings. Then, volatile compositions from 14-d-old aCO_2_ and eCO_2_ barley seedlings were investigated by GC-MS. While 16 VOCs were identified from aCO_2_ barley seedlings, only 9 VOCs were found from eCO_2_ barley seedlings. At last, we discussed the potential role of these chemicals observed during choice bioassays. Our findings lay foundation for functional response of corn leaf aphid under climate change through host plant modifications.

## 1. Introduction

Since the industrial revolution, the atmospheric concentration of carbon dioxide (CO_2_) has been steadily rising from approximately 280 ppm to 401 ppm (Mauna Loa Observatory: NOAA-ESRL) worldwide. Forecasts suggest that the concentrations could double by the year 2100 [1]. As CO_2_ is a substrate for plant photosynthesis, an increase in its concentration in the atmosphere directly impacts plant growth and composition [2,3,4,5]. Hence, the carbon:nitrogen (C:N) ratios of plants increase with the concentration of CO_2_ [2,5,6,7,8,9], which enhanced the photosynthetic rate of C_3_ plants, such as wheat (*Triticum aestivum* L.) and barley (*Hordeum vulgare* L.) [10,11,12,13]. In addition, the alteration of secondary plant chemistry by CO_2_ rise was documented [14,15,16,17]. Indeed, plant grown under eCO_2_ condition usually elicits the production of phenolic compounds, tannins, and flavonoids and suppresses the production of terpenoids [17,18]. However, no consistent trend has been found in the response of insects to these allelochemicals adaptation [19].

Most of herbivorous insect species rely on olfactory signals from their environment to find a mate and to locate a host plant [20]. The VOCs from plants range from fatty acid derivatives, terpenoids, and sulfur compounds to phenylpropanoids [21]. The composition and amounts of plant VOCs can vary depending on several parameters: Plant taxon [22], stage of development [23], physiological status [24], and environmental stresses [25]. However, the effects of increasing the concentration of CO_2_ on the emissions of plant VOCs are not well-defined [26].

Aphids (Hemiptera: Aphididae) are the most important pest insects under temperate climate [27]. They are responsible for the transmission of more than 50% of insect-transmitted plant viruses [28]. Many aphid species produced two types of morphs: A winged (alate) morph, that is mainly responsible for the dispersal and the colonization of new plants, and an wingless (apterous) morph, that mostly stays on the plant on which it was born [29]. Aphids use olfaction to recognize plant hosts from non-plant hosts, which allows them to determine the suitability of different plants [30,31]. Moreover, the variation in behavioral responses to volatiles can also be found in winged and wingless morphs [32]. Volatile blends, based on headspace collections from wheat and oat (*Avena sativa*) plants, elicited similar behavioral responses from both morphs of *R. padi* in olfactometer studies. When compounds were tested individually, the two morphs responded differently [33]. Winged aphids were only attracted/arrested by four of the compounds, whereas wingless morphs responded to 11. However, studies dealing with the behavior of aphids, exposed to VOCs of host plants, grown under ambient and elevated atmospheric CO_2_, are not widespread [34,35,36,37].

As a worldwide pest insect, corn leaf aphid, *Rhopalosiphum maidis* (Fitch) (Hemiptera: Aphididae) caused significant damage on cereal crops, such as barley, corn, wheat, and broad bean [38]. Corn leaf aphid is also a vector of plant viruses including sugarcane mosaic virus (SCMV) and maize dwarf mosaic virus (MDMV), which result in serious damage [39,40,41]. This work aims to investigate the effects of elevated CO_2_ concentrations on foraging behavior of corn leaf aphids. The foraging behavior of aphids to chemical cues of host plants was assessed by using Y glass tube olfactometer. Then, volatile organic compounds from isolated barley seedlings *Hordeum vulgare* L. reared under ambient CO_2_ (aCO_2_), and elevated CO_2_ (eCO_2_) conditions were analyzed by GC-MS.

## 2. Material and Methods

### 2.1. CO_2_ Condition Chambers

Six chambers (60 cm in length, 50 cm in width, and 50 cm in height, PLEXIGLAS^®^ GS, clear 0F00 GT, 8 mm thick; Evonik Industries, Essen, Germany) were used for rearing plants and insects under two different concentrations of CO_2_. In each chamber, a constant airflow (30 L·min^−1^) was pushed through an air pump (Koi flow 30; Superfish, Netherlands). Two levels of CO_2_ concentrations were applied: Ambient level (aCO_2_, 450 ± 50 ppm) and elevated level (eCO_2_, aCO_2_ + 350 ppm) by using a CO_2_ gas tank (>99% purity; Airliquide^®^, Paris, France). Three chambers were used for each CO_2_ treatment. These chambers were maintained at 23 ± 1 °C and 65 ± 10% relative humidity (RH), with a 16:8 h (L:D) photoperiod. Carbon dioxide concentrations, temperature, and RH were continuously monitored in each chamber with MCH-383 SD data loggers (Lutron, Taipei, Taiwan).

### 2.2. Plant Material

Barley, *Hordeum vulgare* L., Etincel cultivar was sown in single plastic plots (7.5 cm diameter, 9.0 cm high), with 25 to 30 seedlings per pot. After sowing, these pots were introduced in aCO_2_ and eCO_2_ chambers separately. Two weeks old barley seedlings (decimal code 12 [42]) were used for host finding behavior tests and volatile analysis.

### 2.3. Aphid Rearing

A colony of Corn leaf aphid, *Rhopalosiphum maidis*, was originally collected from a corn field in the experiment station of Chinese Academy of Agricultural Sciences (39°30′42″N, 116°36′7″E) in Hebei Province, China, was maintained under ambient CO_2_ concentration at a constant temperature of 23 ± 1 °C, 65 ± 10% RH and a photoperiod of 16:8 h (l:d). The colony was reared on barley seedlings in a cage (36 cm in length, 27 cm in width, and 28 cm in height).

To ensure the experiments remained uniform, numerous apterous reproductive adults were transferred to new pots. After 24 h, the adults were removed from the plants, and their offspring were reared on the barley seedlings. Three days old nymphs and eight days old winged and wingless adults were used for the host finding behavior tests.

### 2.4. Foraging Behavior Bioassay

A two-arms glass olfactometer (Y tube olfactometer) was used to investigate the behavioral response of nymphs, winged, and wingless adults to different olfactory stimuli from barley grown under different concentration of carbon dioxide (CO_2_) (eCO_2_: 800 ± 50 ppm; aCO_2_: 450 ± 50 ppm). The aphids were offered one of the following three odor source combinations: Control (clean air) versus aCO_2_ barley seedling, control versus eCO_2_ barley seedling, or eCO_2_ barley seedling versus aCO_2_ barley seedling.

All trials were conducted at 23 ± 1 °C in an observation chamber (60 cm in length, 50 cm in width and 40 cm in height) lightened with three 16-W cool white fluorescent lights, which provided uniform lightening. The main arm of Y-olfactometer (15 cm long and 1.5 cm I.D.) and the two arms (20 cm long and 1.5 cm I.D.) were made of glass. Three black lines (two centimeters from the bottom of the stem or two arms) were drawn on the stem and two arms of Y tube olfactometer separately in order to observe the position of aphids. Plants grown under elevated or ambient concentrations of CO_2_ in glass pot were placed into sealed glass chamber (4 L, 20 cm I.D.) (Analytical Research Systems, Gainesville, FL, USA) and randomly connected to each arm of the Y-olfactometer with Teflon^®^ pipes. A push pump system (PVAS11; Volatile Assay Systems^®^, Rensselaer, NY, USA) was connected to each chamber to carry volatile organic compounds (VOCs) released by barley to the Y-olfactometer. The air was first purified through a charcoal filter to avoid any outdoor contamination. The pushed air flow was kept at 0.7 L·min^−1^.

Aphids were individually placed at the entrance of the stem part, alternating with nymphs, wingless and winged adult aphids. Each insect was allowed to spend five minutes in the Y tube olfactometer. In total 180 aphids were tested for each life stage. The host finding behaviors of aphids were visually observed and simultaneously encoded using The Observer 5.0 software (Noldus^®^, Wageningen, The Netherlands). The following behaviors were recorded during the experiment:

No response: When aphids stayed at the entrance, they did not cross the black line marked on the stem part.

Only searching: When aphids crossed the black line marked on the stem, but did not cross the black line marked on the chosen arm.

Selection: When aphids made a choice and crossed the black line marked on the arm of the Y tube olfactometer.

Between each experiment, new plants were introduced to the chambers. The Y-olfactometer were cleaned with pure n-hexane (>99.7%; VWR^®^, Radnor, PA, USA) and dried at room temperature for about five minutes after testing 15 aphids. Moreover, the chambers and all of the Teflon pipes were washed with n-hexane (>99.7%; VWR^®^, Radnor, PA, USA).

### 2.5. Headspace Analysis of Volatiles from Plants by GC-MS

The upper seedling parts (about 12 cm in length) of aCO_2_ and eCO_2_ barley were carefully sealed in the bell-like glass collection chamber (2 L) separately. To avoid volatile contamination, the base root parts were wrapped with aluminum foil and placed in a cleaned glass bottle. Headspace volatiles from aCO_2_ and eCO_2_ barley seedlings were collected using a dynamic ‘push–pull’ pump system. The pushed airflow was set at 0.7 L·min^−1^ and the pulled air flow was set at 0.3 L·min^−1^. The air entering into the chamber was cleaned by an activated charcoal filter. A 60 mg Tenax TA^®^ thermodesorption tube (Gerstel, Germany), which is made of a microporous polymer of 2,6-diphenylen oxide, was placed at the exit of the glass chamber to trap the volatile compounds carried by the air pulling from the chamber. The tubes were previously cleaned by a thermal conditioner (TC2, Gerstel, Mülheim an der Ruhr, Deutschland), for a period of 11 h at 300 °C. Volatile collection took place over a 24-h period. Straight after volatile collection, the entire aerial portion of the plants was removed to determine dry weight. It allowed the calculation of the amount of VOCs in nanogram per gram of above-ground dry plant. Six replicates were conducted for each condition of CO_2_ concentration of growing, along with the same number of controls (only soil and glass pots wrapped with aluminum).

The volatiles were analyzed by Gas Chromatography coupled with a Mass Spectrometer (GC-MS) (model 7890A; Agilent Technologies Inc., Santa Clara, CA, USA). In this system, the Tenax TA cartridge was thermally desorbed (Thermal Desorption Unit, Gerstel, Mülheim an der Ruhr, Deutschland) at 250 °C for 10 min prior to the injection. In each sample, one microliter of butylbenzene (2.15 ng/μL) was injected as an internal standard.

The entire sample was injected in a HP-5 capillary column (5% Phenyl Methyl, 30.0 m, internal diameter: 0.25 mm, thickness: 0.25 μm, Agilent Technologies^®^, Santa Clara, CA, USA). The carrier gas used was Helium (Initial flow: 1.5 mL/min, Post flow: 0.4 mL/min). The temperature program started at 40 °C for 2 min, and was increased at 4 °C min^−1^ to 95 °C, and then increased at 6 °C min^−1^ to 155 °C for 10 min, and was finally increased at 25 °C min^−1^ to 280 °C hold for 5 min. The detected peaks were identified based on their mass spectrum by using spectral libraries, Pal 600k and Wiley 275 (the MS spectra match factor was minimum 70%).

### 2.6. Statistical Analyses

Binomial proportion tests (equal distribution hypothesized) were used to compare the foraging behavior of nymphs, wingless and winged aphids in Y tube olfactometer. The residence time of each choice was subjected to an analysis by using a general linear model (GLM). The treatment means were compared using the Tukey’s multiple range tests to determine significant difference at a 95% confidence level. Plant VOCs between two CO_2_ levels were tested with independent samples t-test. All analyses were performed using SAS version 9.2 (SAS Institute, Cary, NC, USA).

## 3. Results

### 3.1. Foraging Behaviors of Aphids

**Figure 1:**
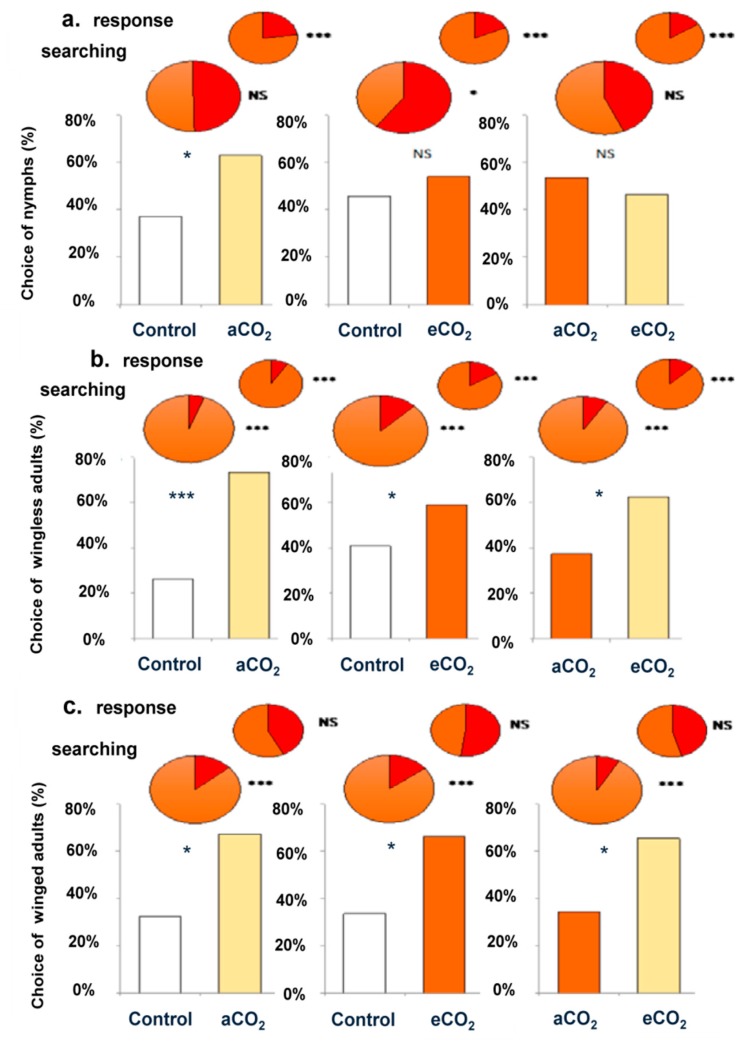
Foraging behavior (in %) of nymph (**a**), wingless (**b**) and winged (**c**) corn leaf aphid during three dual choices including control, aCO_2_ and eCO_2_ barley seedling combinations. Response and searching status assessment corresponded to mobility in the first 2 cm, and before the split of the olfactometer 2 arms, respectively. Red color in pies was negative behaviors (For example, orange color in pies pointed to response, and red color pointed to no response). There were three replicates for each treatment, and a total of 180 aphids were tested. *, *** and NS for *p* ≤ 0.05, *p* ≤ 0.001 and not significant at α = 0.05, respectively.

We tested the foraging behavior of corn leaf aphid for three developmental stages (Figure 1), according to different dual choice, namely control versus aCO_2_ barley seedling, control versus eCO_2_ barley seedling and eCO_2_ versus aCO_2_ barley seedlings. The winged and wingless aphids were more attracted by odors of aCO_2_ barley seedlings when tested in combination with control air or in combination with eCO_2_ barley seedlings. However, nymphs were only attracted by aCO_2_ barley seedlings when it was tested in combination with control, otherwise no significant difference was observed when eCO_2_ was tested in combination with aCO_2_ or with control.

### 3.2. Residence Duration for Searching and Selection Behaviors

**Figure 2:**
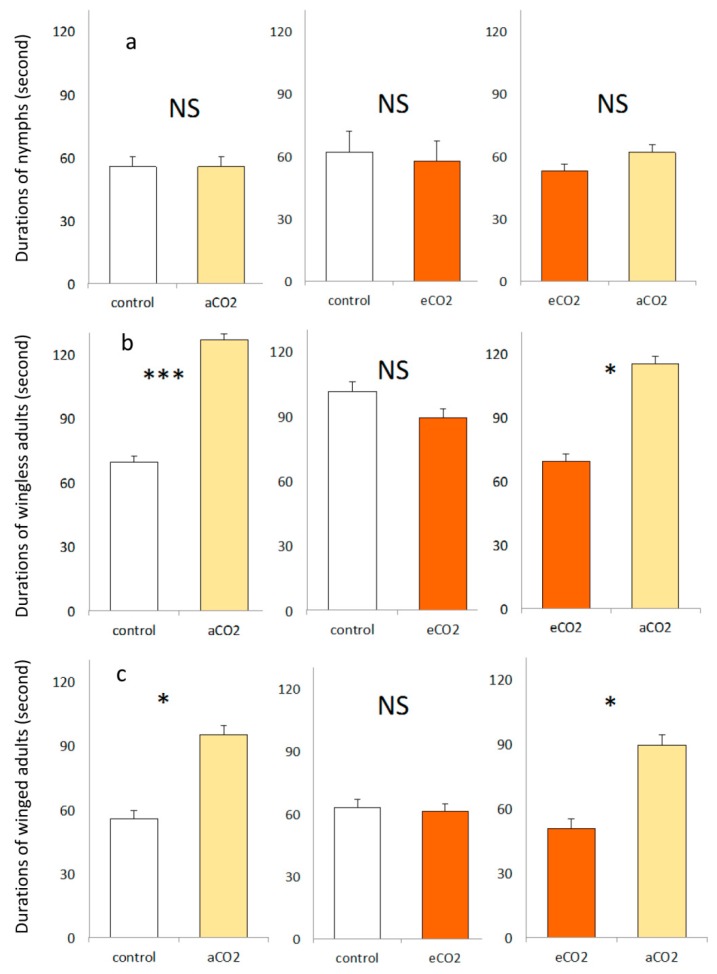
(mean ± se) of nymph (**a**), wingless (**b**) and winged (**c**) corn leaf aphid during three dual choices including control, aCO_2_ and eCO_2_ barley seedling combinations. There were three replicates for each treatment, and a total of 180 aphids were tested. *, *** and NS for *p* ≤ 0.05, *p* ≤ 0.001 and not significant at α = 0.05, respectively.

The typical behavior of nymphs in Y tube olfactometer consisted mainly of searching activities with more than 38% of experimental time. Also, more than 33% of experimental duration of winged aphids corresponded to no response to odor sources, being stationary at the entrance of the olfactometer stem. When the aphids made a choice, the residence duration on each arm was largely affected by kind of life stages (Table 1 and Figure 2). The wingless and winged aphids spent significantly more time in the arms of aCO_2_ barley seedlings when tested against control or eCO_2_ barley seedlings. However, the residence duration of three life stages did not show any significant difference between control versus eCO_2_ barley seedlings.

### 3.3. Volatiles Analysis

**Figure 3:**
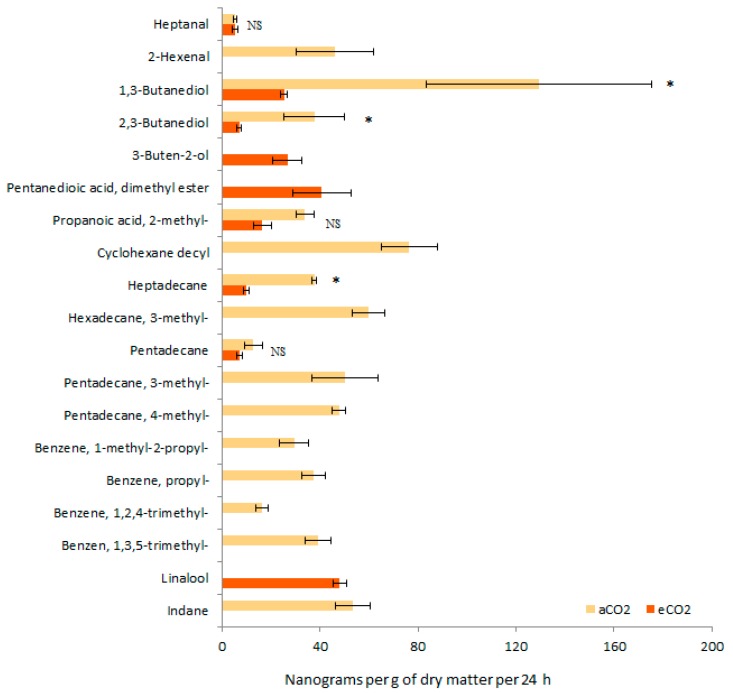
Diversity and abundance of volatile emission (mean ± se in ng per g of dry matter per 24 h) from aCO_2_ and eCO_2_ barley seedling (n = 6 replicates). * and NS for *p* ≤ 0.05 and not significant at α = 0.05, respectively.

According to the GC-MS analysis, 16 and 9 VOCs were identified in aCO_2_, and eCO_2_ barley seedlings, respectively (Figure 3). While, 1,3-butanediol was the main volatile compound emitted by aCO_2_ barley seedling, linalool was the most abundant volatile compound emitted by eCO_2_ barley seedling. Six volatiles were found in both aCO_2_ and eCO_2_ barley seedlings, including heptanal, 1,3-butanediol, 2,3-butanediol, 2-methyl-propanoic acid, heptadecane, and pentadecane. However, the relative abundances decreased in eCO_2_ barley seedlings. The volatile pattern from aCO_2_ barley seedlings was more diversified, including seven supplementary volatiles, namely 2-hexenal, cyclohexane decyl, 3-methyl-hexadecane, 3-methyl-pentadecane, 4-methyl-pentadecane, 1-methyl-2-propyl-benzene, propyl-benzene, 1,2,4-trimethyl- benzene, 1,3,5-trimethyl-benzene, and indane.

## 4. Discussion

In the study, aphid foraging behaviors were found to be influenced by host plants reared in different CO_2_ concentrations. The diversity and abundance of plant VOCs were also differently induced by elevated CO_2_ when compared with aCO_2_.

In our experiment, the wingless and winged aphids were more attracted to the odors of aCO_2_ barley seedlings when tested against eCO_2_ barley seedlings or control. They spent more time during the dual choice on aCO_2_ barley seedlings. However, nymphs were only attracted by aCO_2_ when it was tested in combination with the control. There was some evidence that aphids can detect a variety of individual plant odor components using the hairs on the tips of antennae [43] or that a sensilla at the tibia-tarsus junction may respond to non-volatile chemicals [44]. In aphids, the semio-chemicals are perceived by sensory structures called rhinaria, that are classified in two main groups: Primary and secondary rhinaria. For example, distal (DPR) and proximal (PPR) primary rhinaria allow all morphs and life stages of *Aphis fabae* Scopoli to detect 2-hexenal, a common volatile of their host-plants [45] that is not detected by secondary rhinaria. PPR are usually associated with the perception of host and non-host volatile chemicals, and DPR are probably involved in the perception of the alarm pheromone [30,46].

GC-MS analysis showed that volatiles come from barley seedlings, including aldehydes, alcohol compounds, acid compounds, alkanes, phenyl compounds and others. We found 2-hexenal in the volatile blends of aCO_2_ barley seedlings. Previous study has proved that it is attractive to pea aphid, *Acyrthosiphon pisum* Harris [47]. In our experiment, an amount of linalool was emitted by eCO_2_ barley seedling, which had showed a repellent effect on green peach aphid, *Myzus persicae* (Sulzer) [48], corn leaf aphid, *R. maidis,* and bird cherry-oat aphid, *R. padi* [49]. Therefore, the presence of those compounds in the odor blends of barley, could explain the preference of aphids towards odors of aCO_2_ barley.

The volatiles emitted by barley aerial parts differ both qualitatively and quantitatively, probably because of the experimental treatments or plant stage. Bukovinszky et al. (2005) analyzed the headspace volatiles of 3–4 week old barley; they detected 15 compounds and pointed the volatile profile of barley had the greatest dissimilarity [50]. Wenda-Piesik et al. (2010) collected 11 different volatiles from third leaf stage uninfested barley [51]. Piesik et al. (2010) tested six-week barley, identified about 19 volatiles, and mentioned that mechanical injury and insect feeding caused barley to quantitatively release the highest total VOC concentrations after injury [52]. In our study reported here in, we used the intact barley seedling after 14 days of growth, and identified 16 VOCs in aCO_2_ barley seedlings, and 9 VOCs in eCO_2_ barley seedlings. The barley seedlings were so young and not infested by insects or any fungus, which is probably the reason why we collected less volatiles compared to other research.

The common feature of wingless and winged aphids in the foraging behaviors in the study was that, once they had a response to the volatiles of the plant, their search time was relatively short and they could make a choice quickly. The average time of response at the entrance of Y tube for winged aphid was longer than wingless. The sensitivity to the plant volatiles and the variation in behavioral responses were partly as a result of differences in morphs. Walking is the main way of wingless aphids expanding to nearby plants in the field. When induced by host-plant odors, the wingless aphids will actively walk towards the odor source in the absence of other host cues [53,54]. Winged morphs are capable of making targeted landings on plants under low wind conditions [55,56,57]. However, winged aphids will not attempt to fly without a certain speed of wind prevails [58,59], which indicate that wind probably is an important precondition for the movement of winged aphid. Behavior. The results showed that the nymphs spent more time searching around in the central tube of the olfactometer compared to adults, perhaps they spend more energy moving than adults because of their slower walking speed [60,61,62].

## 5. Conclusions

The aphid behavioral response to plant VOCs is complex. Presented as a blend, these volatile compounds may be integrated as host cues, leading to aphid attraction/arrestment toward the odor source [63]. Every compound of the constitutive blend is going to present individually in olfactometric bioassays to test which one have effect on aphid responses. Future work may focus on how aphids gain information on the identity and quality of a plant from the composition of its volatile blend, and how the interactions between volatiles can affect aphid behavior in changing climatic conditions.

## Figures and Tables

**Table 1:** Summary of a general linear model (GLM) analysis of the effect of life stages (nymphs, wingless and winged adults) and choices on residence duration of *Rhopalosiphon maidis* in Y tube olfactometer, during three dual choices including control, aCO_2_ and eCO_2_ barley seedling combinations.

Source	Model	DF	χ^2^	*p*
Control vs aCO_2_	Life stages	2	60.03	<0.0001
	Choices	1	124.81	<0.0001
	Life stages ^*^ Choices	2	22.83	<0.0001
Control vs eCO_2_	Life stages	2	48.43	<0.0001
	Choices	1	2.62	0.1063
	Life stages ^*^ Choices	2	0.79	0.4557
eCO_2_ vs aCO_2_	Life stages	2	50.60	<0.0001
	Choices	1	130.45	<0.0001
	Life stages ^*^ Choices	2	13.37	<0.0001

“*” pointed to interaction effects between two variables.
